# Clinical audit in European radiology: current status and recommendations for improvement endorsed by the European Society of Radiology (ESR)

**DOI:** 10.1186/s13244-023-01414-9

**Published:** 2023-04-28

**Authors:** David C. Howlett, Paulette Kumi, Roman Kloeckner, Nuria Bargallo, Bettina Baessler, Minerva Becker, Steve Ebdon-Jackson, Alexandra Karoussou-Schreiner, Christian Loewe, Marta Sans Merce, Marta Serrallonga-Mercader, Vasilis Syrgiamiotis, European Society of Radiology

**Affiliations:** 1 Eastbourne Hospital, King’s Drive, Eastbourne, BN21 2UD East Sussex UK; 2 University Hospitals Sussex NHS Trust, Worthing, UK; 3 Institute of Interventional Radiology, University of Lübeck, Lübeck, Germany; 4 Hospital Clinic de Barcelona, Barcelona, Spain; 5 University Hospital Würzburg, Würzburg, Germany; 6 Geneva University Hospital, Geneva, Switzerland; 7 Directorate CRCE, Public Health England, London, UK; 8 Ministry of Health Luxembourg, Luxembourg, Luxembourg; 9 Medical University of Vienna, Vienna, Austria; 10 Hospital Universitari de Vic, Barcelona, Spain; 11 General Childrens Hospital of Athens Agia Sofia, Athens, Greece; 12 Am Gestade 1, Vienna, Austria

**Keywords:** Clinical audit, Radiography, Radiation protection, Patient care

## Abstract

Clinical audit is an important quality improvement activity and has significant benefits for patients in terms of enhanced care, safety, experience and outcomes. Clinical audit in support of radiation protection is mandated within the European Council Basic Safety Standards Directive (BSSD), 2013/59/Euratom. The European Society of Radiology (ESR) has recognised clinical audit as an area of particular importance in the delivery of safe and effective health care. The ESR, alongside other European organisations and professional bodies, has developed a range of clinical audit-related initiatives to support European radiology departments in developing a clinical audit infrastructure and fulfilling their legal obligations. However, work by the European Commission, the ESR and other agencies has demonstrated a persisting variability in clinical audit uptake and implementation across Europe and a lack of awareness of the BSSD clinical audit requirements. In recognition of these findings, the European Commission supported the QuADRANT project, led by the ESR and in partnership with ESTRO (European Association of Radiotherapy and Oncology) and EANM (European Association of Nuclear Medicine). QuADRANT was a 30-month project which completed in Summer 2022, aiming to provide an overview of the status of European clinical audit and identifying barriers and challenges to clinical audit uptake and implementation. This paper summarises the current position of European radiological clinical audit and considers the barriers and challenges that exist. Reference is made to the QuADRANT project, and a range of potential solutions are suggested to enhance radiological clinical audit across Europe.

## Key Points

- Clinical audit is an important component of effective clinical governance and improves patient experience, safety and outcomes.
- The European Council Basic Safety Standards Directive (BSSD), 2013/59/Euratom, mandates clinical audit in support of radiation protection.
- There is persisting variability in clinical audit activity and BSSD awareness across Europe.
- QuADRANT was a project on behalf of the European Commission overviewing current European clinical audit status.
- This paper overviews ESR clinical audit initiatives including the ESR-led QuADRANT project.

## Patient summary

Clinical audit is a quality improvement tool involving systematic review of clinical practice against agreed standards and modifying practice where required. Clinical audit, when implemented effectively, has a significant positive impact on patient care and outcomes.

The European Council Basic Safety Standards Directive (BSSD), 2013/59/Euratom, lays down standards for radiation protection and is designed to ensure the safety of patients and staff involved in medical ionising procedures. The BSSD mandates clinical audit activity in facilities involved in medical ionising exposure and it is a legal requirement that radiology departments (also radiotherapy and nuclear medicine) undertake BSSD-related clinical audit.

The ESR, along with other European organisations (including the European Commission), has introduced a range of clinical audit-related initiatives to support European radiology departments. There is however persisting variation in clinical audit uptake and implementation across Europe.

QuADRANT was a multi-society project, led by the ESR, on behalf of the European Commission, which ran from 2020 to 2022. QuADRANT aimed to review clinical audit activity across Europe, identify existing barriers to clinical audit and to make recommendations for improvements.

This paper reviews existing initiatives in support of radiological clinical audit and considers a range of potential solutions to improve clinical audit uptake and implementation. QuADRANT is an important step forward for clinical audit and for patients and will help support European radiology departments integrate clinical audit into their working practice.

## Introduction

**Fig. 1:**
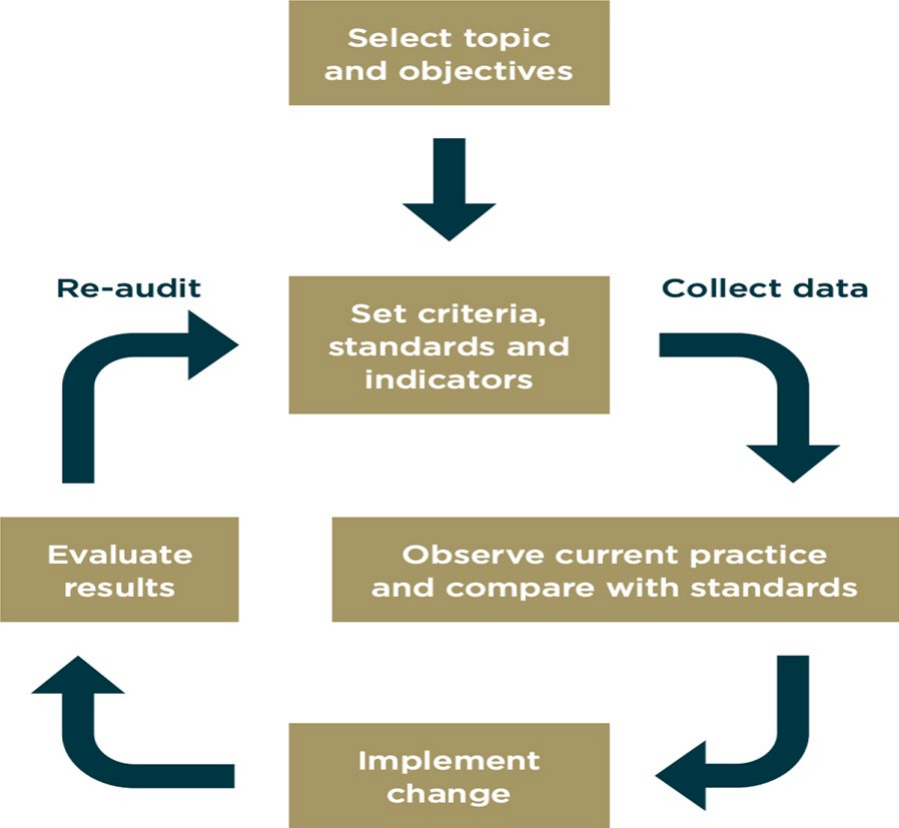
Audit cycle

Clinical audit is recognised as an important quality improvement tool in modern healthcare systems and is an integral component of effective clinical governance [1]. Clinical audit seeks to measure a clinical outcome or process against well-defined standards, established using evidence-based medicine. If standards are not achieved, the reasons for these are evaluated and necessary changes are implemented, with subsequent re-audit to ensure improvement has occurred, the so-called audit cycle [2] (Fig. 1). Clinical audit should be a continuous process with cyclical audit and re-audit.

Implementation of clinical audit practices and processes has well-established benefits in terms of enhanced patient care and service delivery [3]. Healthcare professionals also benefit from involvement in clinical audit through increased professional satisfaction and knowledge and improved communication. In recognition of the importance of clinical audit in improving patient safety and outcomes, clinical audit activity “according to national procedures” is mandated within the European Council BSSD, 2013/59/Euratom [4].

The BSSD lays down standards for radiation protection and was required to be transposed into European Union Member State legislation by February 2018. The BSSD is of particular relevance to all facilities utilising medical ionisation procedures, including European radiology departments. It is recognised, however, that clinical audit in support of radiation protection is most effective when embedded within a wider healthcare clinical audit system. Previous work by the European Commission in 2007/2008 identified variable and often minimal implementation of clinical audit practices across the Member States, and in response, the Commission published a guidance document on clinical audit as part of its Radiation Protection Series [5]. Despite legal requirements and promotion activities, subsequent works by the European Commission [6] and national professional societies, notably the ESR [7–9], have demonstrated persisting variation in European clinical audit uptake and a lack of awareness of BSSD clinical audit requirements. An ESR survey in 2018 [7] sent to all European national radiological societies demonstrated an appreciation of the importance of clinical audit in many national societies but highlighted deficiencies in the infrastructure and resources required for clinical audit to be embedded effectively. A follow-up survey by the ESR in 2021 involving European radiology departments confirmed persisting variability in compliance with the BSSD requirements in relation to clinical audit, following a pilot survey in 2019 [8, 9].

This paper aims to identify existing challenges and barriers to European clinical audit uptake and implementation with an emphasis on radiology departments, and considers both existing initiatives and also proposed developments and recommendations aiming to improve the situation.

### Clinical audit: key definitions

One of the reasons suggested for a lack of engagement in clinical audit is a persisting element of confusion in relation to types of clinical audit and how these differ from regulatory audit and the regulatory process of inspection. A brief summary of key descriptions is included below, and these are covered in more detail in the European Commission Radiation Protection 159 document [5] and publications by HERCA [10, 11].

- Self-assessment/evaluation—a step in preparation for internal clinical audit.
- Internal audit—audit occurring at a local level (individual, departmental, hospital) consistent with national requirements to ensure that clinical practice reflects the policies and procedures of the employer and complies with existing standards.
- Internal audit with external direction—a system whereby guidance/direction is provided by an external body (such as a national professional society) allowing potential coordination of audit across multiple departments and hospitals.
- External audit—an external auditing team (ideally comprising healthcare professionals and including those from the specialty being audited) working across a number of departments/hospitals within a region or country.
- Regulatory audit—verifies that practice is compliant with European Council BSSD regulations and ensures radiation protection-related practices correctly reflect the policies of the employer.
- Inspection—carried out by, or for, a national competent authority to verify compliance with national legal radiation protection requirements, including those addressing the need for clinical audit.

### QuADRANT: a European commission project on clinical audit

QuADRANT represents an important piece of work, led by the ESR on behalf of the European Commission and with several key objectives in relation to European clinical audit uptake and implementation.

(i) To provide an overview of clinical audit activity across Europe with an emphasis on radiation protection.
(ii) To identify good practices and resources in clinical audit and opportunities for cross-pollination of ideas and sharing of resources.
(iii) To identify barriers and challenges to clinical audit uptake and implementation to provide recommendations for improvement.

QuADRANT commenced in January 2020 with a duration of 30 months, and the project was undertaken in partnership with two other professional societies where quality and safety challenges had also been identified, namely ESTRO and EANM. It is beyond the scope of this paper to discuss QuADRANT in detail, but it is important to mention the project in that it highlights many of the issues that are pertinent in relation to variable national and local clinical audit uptake and also identifies potential solutions. The QuADRANT findings have been published in full as part of the European Commission Radiation Protection Series [12] and will also be summarised in a publication in the ESR Insights into Imaging Journal [13].

### Existing clinical audit-related initiatives in radiology

Supporting healthcare practitioners, radiology departments and national radiological professional societies in clinical audit uptake and implementation is a key area of priority for the ESR. The ESR Audit and Standards subcommittee sits within the societal infrastructure with responsibility for promoting and developing clinical audit across the European radiological community, the subcommittee forms a part of the Quality, Safety and Standards Committee which in turn has representation on the ESR Executive Council. The Audit and Standards subcommittee has been involved in a number of significant audit-related initiatives, including the QuADRANT project and the development of Esperanto—the ESR Guide to Clinical Audit in Radiology [14]. The 3rd and revised edition of Esperanto was published in late 2021 containing a review of clinical audit with explanations and definitions and providing a clinical audit toolkit. The toolkit contains a blank template for local radiology department adaptation and then a series (> 60) of bespoke templates on a variety of topics both clinical audit and regulatory audit in nature. Esperanto is open access and has been promoted widely within the ESR membership and can act as a significant support for radiology departments whether early on or more advanced in their clinical audit activity.

It is also worth mentioning in this section a resource developed in the United Kingdom by the Royal College of Radiologists (the national professional society). This is called AuditLive, an open-access, extensive and varied collection of clinical audit templates covering most specialty areas [15]. Both Esperanto and AuditLive represent resources that support clinical audit and have the potential for sharing across countries or adaptation across specialties. Clinical audit guides have been produced by some national professional societies and also other professional bodies [16]. A full list of available resources is available in the QuADRANT publication [12].

The Audit and Standards subcommittee also organises educational sessions on clinical audit and related topics at the annual ESR societal meeting in Vienna, the European Congress of Radiology (ECR), and produces publications and journal articles on clinical audit-related topics [2, 7–9, 17–20]. EuroSafe imaging is another multidisciplinary initiative introduced by the ESR in 2014 to further promote the principles and practice of radiation protection and clinical audit forms an important component [21].

Although it is clear that significant resources have been developed in support of clinical audit in radiology, it is also apparent, as previously alluded to, that resource availability and clinical audit uptake and awareness remain variable and inconsistent across European national radiological professional societies and their constituent radiology departments.

### Barriers and challenges to European clinical audit uptake

The COVID-19 pandemic has provided a significant recent challenge to healthcare systems worldwide, negatively impacting clinical audit investment and uptake. Beyond this, a number of barriers and challenges to clinical audit uptake have been identified, and these were also highlighted as part of the QuADRANT project [3, 12, 22, 23].

Barriers include:

(I) Lack of resourcing (funding) at all levels within the healthcare system.
(II) Low national and hospital priority.
(III) Lack of dedicated time, staff and infrastructure.
(IV) Lack of national/local expertise in audit methodologies.
(V) Ineffective group dynamics and dysfunctional multiprofessional/team working.
(VI) Limited use of clinical audit facilitators/enablers (see later).
(VII) Lack of integration of clinical audit teaching and training into undergraduate and postgraduate education programmes.

### Solutions and recommendations going forwards

Available resources and expertise in support of clinical audit vary across European countries, and variation may also occur within regions and individual hospitals. “One size does not fit all” solutions and recommendations for improving clinical audit uptake and implementation will not be the same uniformly across Europe and need to be adjusted to suit national and local requirements and available resources. Some countries are at the start of their clinical audit journey, whilst others are relatively advanced. Two other themes recurred during the QuADRANT project—firstly the development of a “non-threatening” and “no blame” culture of clinical audit and secondly the concept of “top down, bottom up” in terms of development of clinical audit infrastructure and integration. A top-down improvement relying on national/central audit directives and initiatives, allied with a bottom-up approach, where local healthcare practitioners are empowered to develop clinical audit at the departmental/hospital level, seems to be most effective [24]. Such processes take time to develop and embed, requiring resources and prioritising within the healthcare system.

QuADRANT provided recommendations for improving clinical audit uptake and implementation across Europe, and these are outlined briefly below:

(i) Establishment of a national body responsible for clinical audit development, development of national audit/best practice guides and fostering external European and international relationships. For most countries in Europe, resource allocation to develop national and local audit infrastructure will be necessary. The nature and quantity of additional resources will be guided by national priorities, available expertise and infrastructure. The identification and prioritisation of additional monetary funding in particular is likely to be required to facilitate and embed changes in practice.
(ii) To further promote the BSSD requirements around clinical audit and to ensure that processes of inspection also include clinical audit.
(iii) The national professional societies (including those of radiologists, radiographers and medical physicists) have been identified as potentially pivotal in the development and implementation of effective national audit programmes. Lack of resources was highlighted as a major barrier to national professional society involvement, with investment required to develop: administrative support; information technology systems and communication with membership; data collection mechanisms; and training of auditors. Shortages of healthcare professionals are also an important resource issue in some countries and will need to be addressed. National professional societies are well placed to lead and develop national audit programmes and models of successful involvement do already exist. In the UK, the national professional body, the Royal College of Radiologists, acts as a central hub externally directing internal audit projects via a network of over 200 radiology departments with internal departmental audit leads. In Finland, there is a mature system of external audit led by professionals in the specialty being audited, supported by the national professional society [25]. For most national radiological professional societies, significant development and resource allocation are likely to be necessary, following a process of clinical audit prioritisation at the national level. The QuADRANT publication and recommendations can help facilitate this.
(iv) Implementation of facilitators/enablers of clinical audit where appropriate. For example, direct/indirect remuneration of individuals (salary) or hospitals (budgets); allowing (funded) time for clinical audit in work schedules; enhanced hospital accreditation or healthcare professionals certification where clinical audit activity is demonstrated; enhanced access to staff or equipment; and academic recognition.
(v) Active promotion and integration of clinical audit teaching into radiology-related undergraduate and postgraduate education programmes. Participation in clinical audit should form part of continuing professional development for healthcare professionals. These are areas where the national professional society can input effectively and provide leadership and guidance.
(vi) Enhance and formalise patient involvement in clinical audit policy development and practice.
(vii) Actively share and develop clinical audit resources between countries and specialties and look to encourage links with other European and international agencies.

## Conclusion

Clinical audit is an important component of providing safe and effective modern-day health care, with benefits for both patients and staff. Despite clinical audit being mandated within the BSSD and significant supportive work by European professional societies, radiological clinical audit uptake and implementation remain variable across Europe. Solutions are complex and multifactorial and will require resourcing and effective collaboration between relevant professional bodies and national administrations. The ESR-led project, QuADRANT, on behalf of the European Commission, will provide a key stepping stone in this process.

## Data Availability

Detailed data on the results reported in this article can be found in the form of tables and text in the final published version of the QuADRANT which can be accessed via the European Commission website https://commission.europa.eu/index_en.

## Abbreviations

BSSD: Basic Safety Standards and Directive
EANM: European Association of Nuclear Medicine
ESR: European Society of Radiology
ESTRO: European Society of Radiotherapy and Oncology
HERCA: Heads of the European Radiological Competent Authorities
QuADRANT: Quality Improvement through Clinical Audit in Diagnostic (including Interventional) Radiology, Radiotherapy and Nuclear Medicine (including Therapies)
